# Targeted Analysis of Tears Revealed Specific Altered Metal Homeostasis in Age-Related Macular Degeneration

**DOI:** 10.1167/iovs.63.4.10

**Published:** 2022-04-15

**Authors:** Eva Valencia, Montserrat García, Beatriz Fernández-Vega, Rosario Pereiro, Lara Lobo, Héctor González-Iglesias

**Affiliations:** 1 Ophtalmological Research Foundation, University Institute Fernández-Vega, University of Oviedo, Oviedo, Spain; 2 Department of Physical and Analytical Chemistry, Faculty of Chemistry, University of Oviedo, Oviedo, Spain; 3 Ophthalmological Institute Fernández-Vega, Oviedo, Spain

**Keywords:** age-related macular degeneration, tear, targeted analysis, (metallo-)proteins, metals, dyshomeostasis

## Abstract

**Purpose:**

Specific altered metal homeostasis has been investigated in the tear film of age-related macular degeneration (AMD) patients considering that metal dyshomeostasis contributes to the production of free radicals, inflammation, and apoptosis and results in conformational changes of proteins.

**Methods:**

A multitargeted approach based on spectrophotometry and mass spectrometry techniques has been implemented to the multiplexed quantitation of lactoferrin (LF), S100 calcium binding protein A6 (S100A6), metallothionein 1A (MT1A), complement factor H (CFH), clusterin (CLU), amyloid precursor protein (APP), Mg, P, Na, Fe, Cu, Zn, and Ca, in the tear film from 60 subjects, 31 patients diagnosed with the dry form of AMD, and 29 healthy individuals

**Results:**

Significant up-regulations of MT1A (1.9-fold) and S100A6 (1.4-fold) and down-regulations of LF (0.7-fold), Fe (0.6-fold), Mg (0.7-fold), and Cu (0.7-fold) were observed in AMD patients, when compared to control subjects. Of all the studied variables, only APP showed negative correlation with age in the AMD group. Also, positive correlations were observed for the variables Mg and Na, Cu and Mg, and P and Mg in both the AMD and control groups, whereas positive correlations were exclusively determined in the AMD group for Cu and LF, Na and Ca, and Mg and Ca. The panel constituted of MT1A, Na, and Mg predicts AMD disease in 73% of cases.

**Conclusions:**

The different levels of target metals and (metallo-)proteins in the tear film suggest altered metal homeostasis in AMD patients. These observed pathophysiological changes may be related with the anomalous protein aggregation in the macula.

Aging is an irreversible process inevitably associated with disease, being the predominant risk factor for most conditions that limit health span. According to data from the World Population Prospects 2019,^1^ one in eleven people in the world was over the age of 65 in 2019 (9%), which is expected to increase to one in six people by 2050 (16%). The greater aging of the population turns some diseases from occasional to very frequent, with particular impact in neurodegenerative pathologies. Age-related neurodegenerative diseases are a group of disorders sharing common features and molecular patterns characterized by abnormal accumulation of proteins and selective neuronal degeneration and death.^2^ Insoluble oligomerized protein deposition has been identified within the eye during the onset of age-related macular degeneration (AMD). Actually, AMD is the leading cause of noninherited irreversible vision loss in developed countries and a very prevalent cause of legal blindness in the elderly.^3^ Early AMD initiates with the formation of extracellular deposits, called *drusen*, between the basal lamina of the retinal pigment epithelium (RPE) and the Bruch's membrane, which progressive degeneration leads to the advanced stages of the disease, the neovascular (or wet) or geographic atrophy (or dry) forms of AMD.^4^ Current therapies exist for neovascular AMD, representing 10% to 15% of cases, but there is no approved treatment for dry AMD, accounting for 85% of cases of advanced disease.^5^^,^^6^

Drusen composition is highly complex, containing lipids, minerals, and proteins abnormally aggregated,^7^^–^^11^ the formation molecular mechanisms of which include protein misfolding induced by metals.^12^^,^^13^ The impairment of intracellular homeostatic control of metals underlies their cytotoxic effects, in the oligomerization of proteins and in the aggregation and consequent loss of functionality of molecules. Also, metal dyshomeostasis contributes to the production of free radicals, inflammation, and apoptosis and results in conformational changes of proteins involved in neurodegeneration.^14^^–^^16^ Among others, dyshomeostasis of Zn, Fe, Cu, or Ca induces various detrimental intracellular events, including oxidative stress, DNA fragmentation, protein misfolding, and activation of apoptosis, which leads to neuronal death.^17^^,^^18^ Therefore specifically altered metal homeostasis may occur in AMD disease, at both the local and systemic levels. Considering that is required a major understanding of the effects of metals changes during neurodegenerative processes, the application of innovative analytical and biochemical approaches, including (metallo-)proteomic analysis, may contribute to shed light on metal-protein interactions and their pathogenic effects in AMD.

Although neurodegeneration during AMD occurs in the RPE and neurosensory retina, possible changes could be observed in the anterior segment of the eye globe, specifically in readily accessible fluids with noninvasive sampling such as tear film.^19^ The tear film, produced by Meibomian, lacrimal, and accessory glands, along with goblet cells,^20^ is composed of three layers: the inner hydrophilic mucin layer, the middle aqueous layer, and the outer lipid layer (see Fig. 1).^21^ This thin fluid layer (up to 6 µm thickness) is very promising for the discovery and implementation of biomarkers or to study local alterations related to eye diseases, because it contains a comparatively simple proteome composed of a variety of molecules and removes local waste products, drugs, and disease-related media.^22^ Tear fluid permits a noninvasive procedure for sampling by Schirmer test papers or glass microcapillaries, requiring no incision into the body or tissue removal.^23^ However, the conjunctival sac has a capacity of approximately 15 to 30 µL, and the natural tear film volume is 7 to 8 µL, severely limiting the multitargeted analysis. This is most dramatic when studying the elderly population, because tear production or secretion starts to be impaired.^20^^,^^24^ So far, limited studies have been carried out for the discovery of specific changes in the tear film associated with AMD neurodegenerative progression.^25^^,^^26^ Nevertheless, a major constraint remains in the lack of powerful methodologies for a multidisciplinary approach, because conventional biological/biomedical techniques only address a specific part of the problem, and tear film represents a great challenge. Therefore methods for the quantitative multiparametric analysis of molecules and metals in tear film are currently mandatory. In this work, we have implemented a multitargeted approach based on spectrophotometry and mass spectrometry techniques to decipher possible existing changes in the homeostasis of metals, metalloproteins, and protein-related metals in AMD patients. To this end, quantitative analysis of lactoferrin (LF), S100 calcium-binding protein A6 (S100A6), metallothionein 1A (MT1A), complement factor H (CFH), clusterin (CLU), amyloid precursor protein (APP), Mg, P, Na, Fe, Cu, Zn, and Ca have been carried out in low volume of tears from control subjects and patients diagnosed with the dry form of AMD.

**Figure 1.**
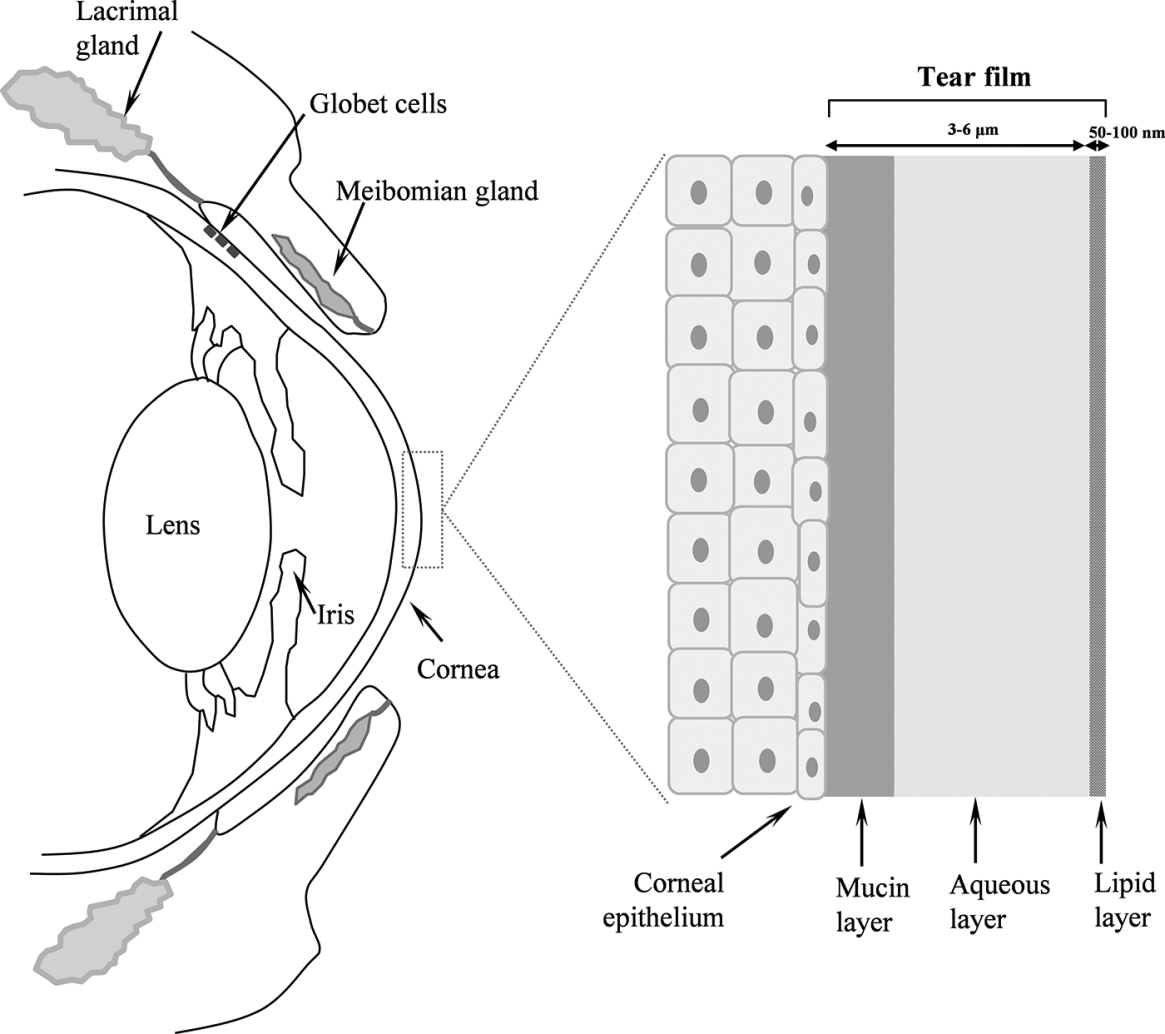
Diagram summarizing the lacrimal apparatus and the tear film structure.

## Methods

### Study Subjects

The present observational, prospective and case-controlled study involved 60 subjects: 31 patients diagnosed with the dry form of AMD and 29 healthy individuals (controls). The study adheres to the tenets of the Declaration of Helsinki on Biomedical Research Involving Human Subjects and was approved by the Clinical Research Ethics Committee at Principality of Asturias (Oviedo, Spain). Study subjects were recruited at the Institute of Ophthalmology Fernández-Vega (Oviedo, Spain) based on the agreed inclusion and exclusion criteria, signed informed consent, and the research was conducted by medically qualified personnel. Complete ophthalmic examinations were performed for patients and controls, including slit-lamp biomicroscopy and funduscopy in both eyes. AMD-diagnosed patients were further examined by fluorescence fundus angiography or optical coherence tomography. Only individuals with dry AMD and evidence of an active form of geographic atrophy in at least one eye and absence of choroidal neovascularization were included. Control subjects were selected from patients over 60 years old and with absence of AMD or glaucoma. Specifically, controls did not show any sign of drusen, including reticular pseudodrusen, or pigment abnormalities in none of the eyes, being in stage 1 of the Clinical Age-Related Maculopathy Staging classification (i.e., no drusen).^27^ The exclusion criteria for both controls and AMD patients was the absence of other relevant ocular pathologies, such as diabetic retinopathy or any ocular surface disease including dry eye syndrome, eye surface disorders, glaucoma, and previous ocular surgery except for cataract extraction. Those patients or healthy subjects using topical medications or contact lens were also discarded to avoid influences in the tear film composition.

### Sample Collection and Preparation

Tear film was collected from each eye, when possible, using calibrated glass microcapillary tubes with 10 µL of capacity (Blaubrand intraMark, Wertheim, Germany). Sample collection was performed in the morning hours, between 9 AM and 12 PM, using sterile gloves. The microcapillaries were placed into the lower conjunctival sacs of each eye avoiding any contact with the ocular surface to minimize its irritation, obtaining the tear samples from the inferior tear meniscus without anesthesia. Once collected, tear samples from each individual eye were immediately placed in Eppendorf tubes using a plastic syringe and stored at −80°C until analysis. The typically collected tear volume reached 1 to 5 µL.

### Molecular Assays

Quantitation of six proteins of interest was performed using commercial ELISAs following the instructions of the manufacturer. Commercially available sandwich-type ELISA kits from Abcam (Cambridge, UK) were used for the quantification of four of the candidate proteins, the human LF (ab200015), the human CFH (ab252359), the human CLU (ab174447), and the human APP (ab216944). Commercial ELISA kits purchased from Abyntek Biopharma (Vizcaya, Spain) and LS BIO (Seattle, WA, USA) were used for the analysis of human S100A6 (ABK1-E1028) and MT1A (LS-F10296), respectively. For APP, CFH and S100A6 quantitation the tear samples were diluted 1:200, for MT1A quantitation the tear samples were diluted 1:1,000, for CLU quantitation the tear samples were diluted 1:6,000 and for the LF quantitation tear samples were diluted 1:1,000,000. The concentrations of targeted proteins were expressed in nanograms of the protein per milliliter of tear for APP, CFH, S100A6, MT1 and CLU, and in milligrams of the protein per milliliter of tear for LF. Representative analytical parameters for each of ELISA assays have been included in Supplementary Table S1.

Total protein concentration in tears was determined using the commercial QuantiPro BCA Assay Kit (Sigma Aldrich, Madrid, Spain). The calibration range was 0 to 50 µg mL^−^^1^ with BSA as standard. The tear samples were diluted 1:2500. A working solution containing copper compound and bicinchoninic acid was added to the samples. The proteins present in tears, reduced Cu^2+^ to Cu^+^ and a complex between copper and bicinchoninic acid was formed, the absorbance of which was measured at 562 nm wavelength. All absorbance measurements were monitored using the spectrophotometer PerkinElmer 2030 Multilabel Reader VICTORTM X5 (Waltham, MA, USA).

### Multielemental Quantitation Using Flow Injection Analysis (FIA) and Inductively Coupled Plasma Mass Spectrometry (ICP-MS) Detection

Multi-elemental analysis of Ca, Mg, P, Na, Zn, Fe and Cu was performed by external calibration using a 7900 ICP-MS (Agilent, Santa Clara, CA, USA) equipped with collision/reaction cell pressurized with He gas (4.3 mL · min^−1^ flow rate) to minimize polyatomic interferences. As internal standard, Ga was used for matrix effect correction. The FIA system, consisting of a six-channel Rheodyne valve 3125 (Rheodyne, Rohnert Park, CA, USA), with a 5 µL loop, was on line coupled to the ICP-MS. A glass syringe of 100 µL volume (Hamilton, Reno, NV, USA) was used to load samples and standards through the system.

The standard stock solution of Mg, P, Fe, Cu, Zn (1,000 mg · L^−1^), Na, Ca (10,000 mg · L^−1^) and Ga (1,000 mg · L^−1^) were purchased from Merck (Darmstadt, Germany) and used for the external calibration curve. The concentration range for calibration was 0 to 200 µg · L^−1^ for Mg and P, 0 to 500 µg · L^−1^ for Na, Zn, and Fe, 0 to 60 µg · L^−1^ for Cu, and 0 to 1 mg · L^−1^ for Ca. Each calibration standard was injected five times. To determine the multielemental concentration in tear film, samples were diluted with 0.14 M HNO3 (trace metal grade; Fisher Scientific, Waltham, MA, USA) using a 1:20 dilution for most of the elements, with the exception of Na requiring a higher dilution of 1:2000. Thus only 1 µL of tear film from each subject was used to determine their multielemental composition.

### Statistical Analysis

Demographic characteristics of AMD patients and control subjects were performed with SPSS Statistics version 15.0 (IBM Corp., Armonk, NY, USA). In the case of quantitative variables (age), significant differences were analyzed using the Mann-Whitney U test, and qualitative variables (gender and the presence of cataracts, arterial hypertension or cardiovascular disease) were analyzed using the χ^2^ test. Comprehensive studies of all obtained data from both elemental and molecular analysis, reported as mean ± standard error of the mean, was carried out using SPSS Statistics version 15.0 and GraphPad Prism version 3 for Windows (San Diego, CA, USA). Normal distribution was evaluated by the Shapiro-Wilk's test, and, subsequently, significant differences between control and AMD groups for proteins and elements were analyzed using the Mann-Whitney U test for those without normal tendency, or Student's *t* test was selected for normal distribution. Correlation analysis was carried out using SPSS Statistics version 15.0. A *P* value <0.05 was considered statistically significant. Further stepwise discrimination analysis was carried out with SPSS using the concentrations of the proteins and elements determined by ELISA and FIA-ICP-MS, respectively, in the different sample groups. Additionally, logistic regression analysis using SPSS software was performed for further control of potential confounders using SPSS.

Several statistical tools based on Machine Learning approaches were applied using Orange Canvas software v2.6 (http://orange.biolab.si) to assess which method provided the best accuracy for the correct classification of samples based on the selected panel of proteins or elements. These tools included receiver operating characteristic curve analyses for each of the markers, naive Bayes, k-nearest neighbor, random forest, classification trees, and support vector machine.^28^

## Results

### Patients

Demographic characteristics of AMD patients and control subjects are collected in Table 1. The population origin recruited in the present study was Caucasian and mostly from the northernmost regions of Spain. No statistically significant differences were obtained for age, gender, the presence of cataracts, arterial hypertension, or cardiovascular disease, when compared dry AMD cases with controls (*P* > 0.05). Statistically significant differences were observed for dyslipidemia in AMD patients when compared to control subjects (*P* = 0.002). The dry AMD group consisted of 31 individuals, comprising 18 women and 13 men, with a mean age of 80.7 ± 8.0 years, whereas the control group included 29 subjects, formed by 18 females and 11 males, with a mean age of 77.2 ± 8.9 years. In both groups, the percentage of cataractous subjects was similar, specifically 75.9% for controls and 77.4% for AMD patients. The significantly higher percentage of individuals with dyslipidemia in the AMD group (45.2%), compared to controls (6.9%), indicates a possible causal role of plasma lipid levels in the pathogenesis of AMD.^29^^,^^30^ Systemic treatments were assessed, including the common oral treatments for both control and AMD subjects (Supplementary Table S2).

**Table 1.** Demographic Characteristics of AMD Patients and Controls

Study	Age	Age	Gender				Cardiovascular
Population (n)	(Mean ± SD)	Range	(Female/Male)	Cataracts	AHT	Dyslipidemia	Disease
Controls (n = 29)	77.2 ± 8.9	63–89	18 (62.1%)/11	22 (75.9%)	13 (44.8%)	2 (6.9%)	2 (6.9%)
AMD (n = 31)	80.7 ± 8.0	62–101	18 (58.1%)/13	24 (77.4%)	14 (45.1%)	14 (45.2%)	3 (9.7%)

n, number of subjects; SD, standard deviation; AHT, arterial hypertension.

### Targeted Molecular Analysis of Tear Film

**Figure 2.**
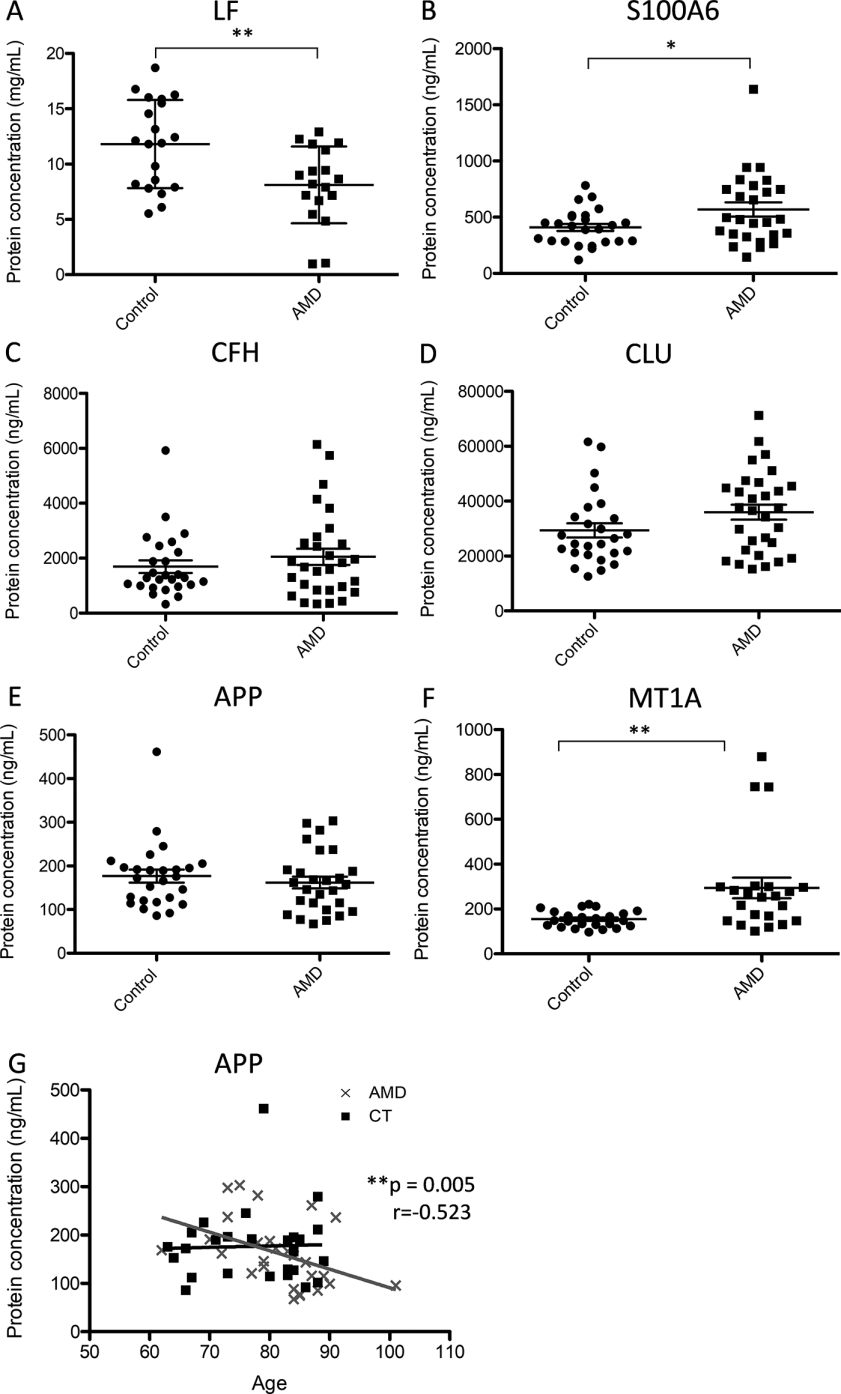
Dot plots of (**A**) LF, (**B**) S100A6, (**C**) CFH, (**D**) CLU, (**E**) APP, and (**F**) MT1A protein concentrations determined in the tear film of dry AMD patients and control subjects. (**G**) Correlation between APP concentrations of AMD patients and control individuals and aging. *r* value, Pearson correlation coefficient. *P value < 0.05; **P value < 0.01; ***P value < 0.001.

Total concentrations of LF, S100A6, CFH, CLU, APP and MT1A were individually quantified in 29 control subjects and 31 dry AMD patients, using ELISA assays (Table 2), dot plots of which are shown in Figure 2. Tear samples from both eyes of same individual were pooled, when possible. Because of the low available sample volume, molecular composition was determined in most, but not all, study subjects. Of all the analyzed proteins, only LF, S100A6, and CLU have been previously quantified in tears.^31^^–^^34^ In our experiments, statistical significant differences were observed for the proteins LF (0.7-fold change, *P* = 0.004), S100A6 (1.4-fold change, *P* = 0.03) and MT1A (1.9-fold change, *P* = 0.006), when comparing AMD patients and control subjects (see Figs. 2A, 2B, 2F). By contrast, no significant differences were observed for CFH, CLU, and APP (Figs. 2C–E; *P* > 0.05). Correlations between the proteins analyzed and the age of the recruited individuals were evaluated using Pearson coefficient, showing only significant negative correlation for APP exclusively in the AMD group (Fig. 2G; *r* = –0.523, *P* = 0.005). Averaged total protein concentration in tears for control individuals reached 8.87 ± 4.84 mg · mL^−1^, whereas for AMD patients it was 9.26 ± 5.28 mg · mL^−1^, with no statistically significant differences observed between groups, being in the range of reference published.^35^

**Table 2.** Concentrations of Proteins and Elements Determined in the Tear Film of AMD Patients and Control Subjects

	Control	AMD	Fold-change	*P* Value
Protein concentrations				
Lactoferrin (average ± SD, mg/mL)	11.81 ± 3.98	8.11 ± 3.47	0.7	0.004
S100A6 (average ± SD, ng/mL)	409.8 ± 158.9	568.59 ± 321.9	1.4	0.03
CFH (average ± SD, ng/mL)	1692 ± 1167	2051 ± 1573	1.2	0.3
Clusterin (average ± SD, mg/mL µg/mL)	29.33 ± 13.01	35.99 ± 14.94	1.2	0.09
APP (average ± SD, ng/mL)	176.71 ± 76.04	161.88 ± 69.86	0.9	0.3
MT1A (average ± SD, ng/mL)	154.45 ± 36.56	293.55 ± 214.00	1.9	0.006
Element concentrations				
Na (average ± SD, µg/mL)	2125 ± 524	1783 ± 678	0.8	0.09
Mg (average ± SD, µg/mL)	9.74 ± 3.68	6.50 ± 3.27	0.7	0.01
P (average ± SD, µg/mL)	3.95 ± 2.25	2.94 ± 2.14	0.7	0.2
Ca (average ± SD, µg/mL)	23.98 ± 9.15	22.46 ± 12.91	0.9	0.7
Fe (average ± SD, µg/mL)	3.53 ± 2.01	2.13 ± 1.37	0.6	0.03
Cu (average ± SD, µg/mL)	0.17 ± 0.07	0.11 ± 0.04	0.7	0.009
Zn (average ± SD, µg/mL)	0.36 ± 0.26	0.27 ± 0.21	0.7	0.3

### Targeted Multielemental Analysis of Tear Film

**Figure 3.**
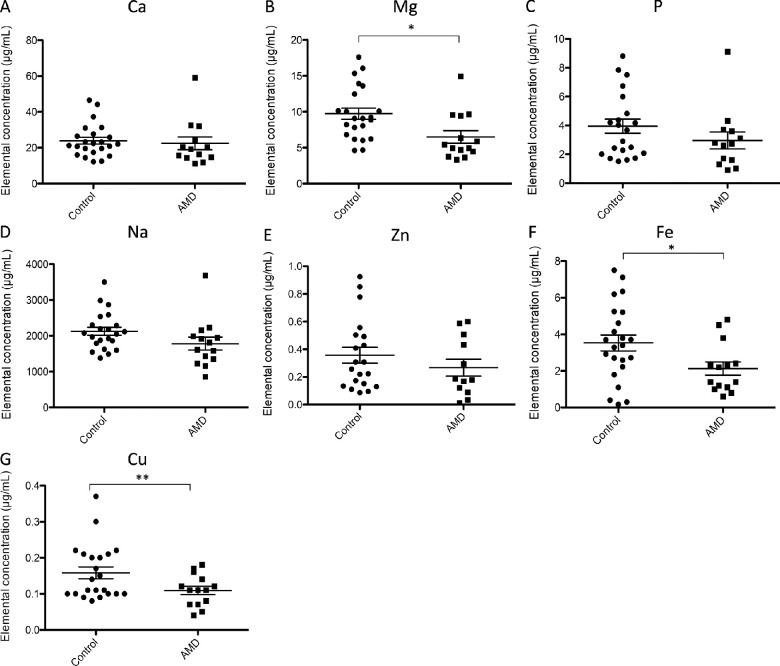
Dot plots of (**A**) Ca, (**B**) Mg, (**C**) P, (**D**) Na, (**E**) Zn, (**F**) Fe, and (**G**) Cu elemental concentrations determined in the tear film of dry AMD patients and control subjects. *P value < 0.05; **P value < 0.01; ***P value < 0.001.

Quantitative multiparametric elemental analysis of Ca, Mg, P, Na, Cu, Fe and Zn was carried out in individual human tears by FIA-ICP-MS (Table 2). Dot plots depicting changes in the concentrations of each element, when comparing AMD and control groups are shown in Figure 3, in which normal distribution and unpaired *t* test was selected to examine possible variations between cohorts. Significant down-regulation was observed for Mg (0.7-fold change, *P* = 0.01, 9.74 ± 3.68 µg · mL^−1^ control group vs. 6.50 ± 3.27 µg · mL^−1^ AMD group), Fe (0.6-fold change, *P* = 0.03, 3.53 ± 2.01 µg · mL^−1^ control group vs. 2.13 ± 1.37 µg · mL^−1^ AMD group) and Cu (0.7-fold change, *P* = 0.009, 0.17 ± 0.07 µg · mL^−1^ control group vs. 0.11 ± 0.04 µg · mL^−1^ AMD group), when comparing AMD patients and control subjects (see Figs. 3B, 3F, and 3G). Correlations between all the elements analyzed and the age of the recruited individuals were also evaluated using Pearson coefficient, showing no statistically significant correlation for none of the variables.

### Study of Variable Associations

**Figure 4.**
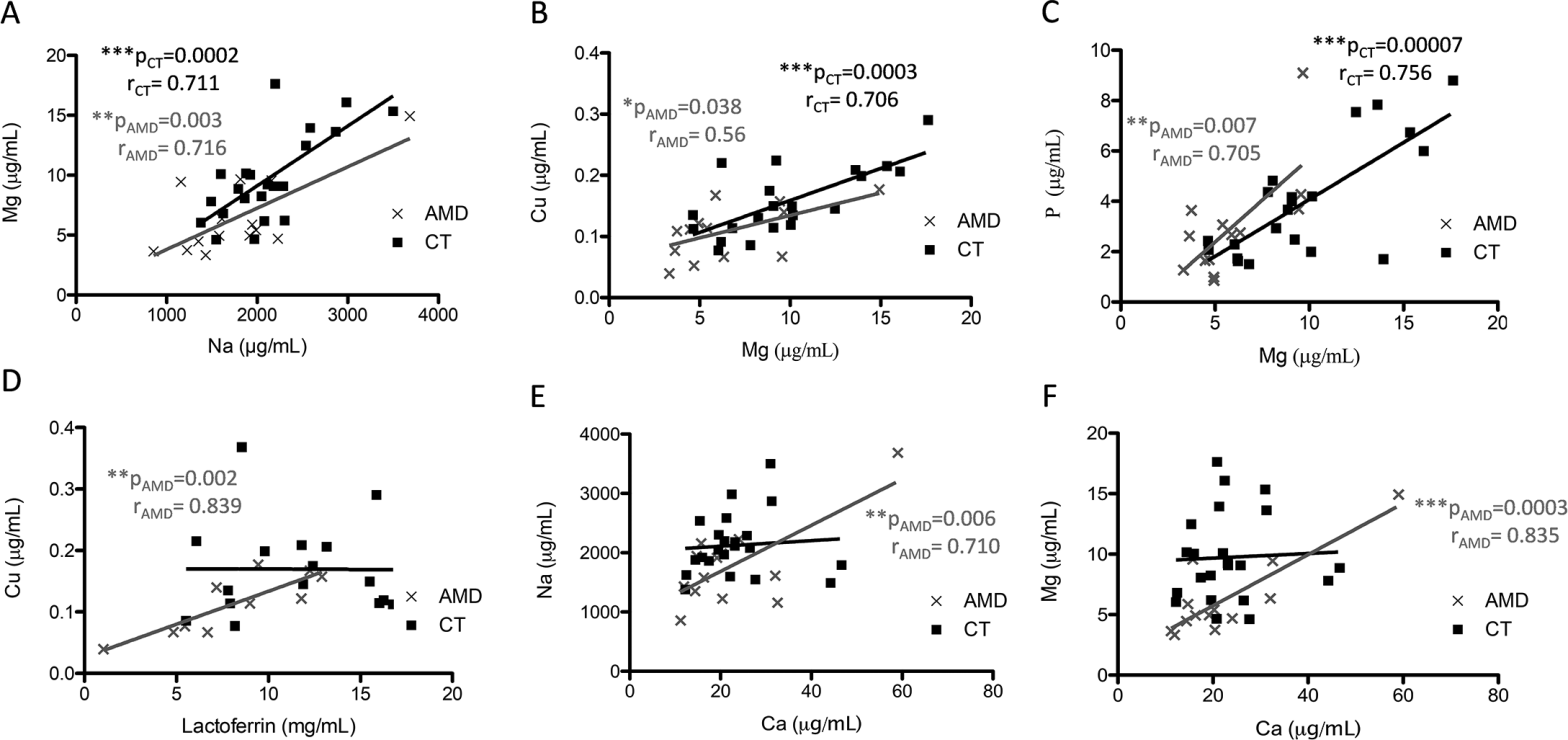
Correlation analysis between (**A**) Na and Mg, (**B**) Mg and Cu, (**C**) Mg and P, (**D**) LF and Cu, (**E**) Ca and Na, and (**F**) Ca and Mg, respectively. *r* value, Pearson correlation coefficient. *P value < 0.05; **P value < 0.01; ***P value < 0.001.

A correlation study among elements and proteins was carried out in AMD patients and control subjects using the Pearson coefficient, shown in Figure 4 the obtained significant associations. Higher negative or positive *r* values indicate the grade of correlation between variables. Positive correlations were observed for both AMD and control groups when association of the variables Mg and Na (*r* = 0.716, *P* = 0.003 in the AMD group; *r* = 0.711, *P* = 0.0002 in the control group), Cu and Mg (*r* = 0.556, *P* = 0.038 in the AMD group; *r* = 0.706, *P* = 0.0003 in the control group), and P and Mg (*r* = 0.705, *P* = 0.007 in the AMD group; *r* = 0.756, *P* = 0.00007 in the control group) were studied (see Figs. 4A–C). Highly significant positive correlations were observed for the AMD group when association of the variables Cu and LF (*r* = 0.839, *P* = 0.002), Na and Ca (*r* = 0.710, *P* = 0.006), and Mg and Ca (*r* = 0.835, *P* = 0.0003) were evaluated (Figs. 4D–F).

### Data Filtering and Logistic Regression

The observed significant differences between AMD and control groups including individuals with dyslipidemia were further explored in a two-step comprehensive statistical analysis to evaluate the influence of this systemic risk factor in the multitargeted results obtained. First, the dyslipidemic patients were excluded and filtered data and parameters were re-analyzed. Total concentrations of LF, S100A6, CFH, CLU, APP, MT1A, Ca, Mg, P, Na, Cu, Fe and Zn obtained for control subjects (n = 27) and AMD patients (n = 17), all of them without dyslipidemia, are shown in Supplementary Table S3. Overall, comparable results to those considering all subjects (Table 2) were obtained, with the exception of Mg. When considering all patients, MT1A, S100A6, LF, Fe, and Cu showed similar fold change (1.9-, 1.4-, 0.7-, 0.6-, 0.7-fold, respectively) to when dyslipidemic subjects were excluded (2.1-, 1.4-, 0.5-, 0.5-, 0.6-fold, respectively), with even smaller *P* values in the latter case (see Supplementary Table S3). Only Mg lost the significant differences when dyslipidemic subjects were excluded (0.7-fold, *P* = 0.07), as compared with unfiltered data (0.7-fold, *P* = 0.01), although the fold-change was the same. This difference in Mg may be related to the lower number on individuals resulted after filtering subjects with of dyslipidemia as a risk factor.

Second, a multivariate logistic regression was carried out in all recruited subjects (31 AMD patients and 29 control subjects), using SPPS with the unfiltered data, to evaluate the role of several confounders, including dyslipidemia, in the differences observed between the AMD group in comparison to control subjects. This analysis confirmed that the risk factor dyslipidemia (*P* = 0.989) did not contribute to the statistically significant differences observed between groups, discarding possible confounders among the demographic conditions and the studied proteins and elements, and therefore the altered levels of MT1A, S100A6, LF, Fe, and Cu in the tear film between both cohorts are result of AMD disease.

### Machine Learning Models

The concentrations determined for the six proteins and the seven elements in tear samples from AMD patients and control subjects were used to perform a stepwise discriminant analysis by SPSS to determine the best variables classifying both groups. The panel consisting of MT1A, Na, and Mg correctly assigns the 89% of the samples group and consequently was used to generate multivariate predictive models. The whole data set (test on train data) was used for training and then for testing with distinct machine learning algorithms, including random forest, naive Bayes, k-nearest neighbor, classification tree, and support vector machine. Later, the classification method was validated using the remaining 30% of data to make predictions on future cases for which the target answer is unknown, providing the correct assignment (CA), sensitivity, specificity, area under the curve, and precision of the model (see Table 3). The best classification efficiency was obtained when using the MT1A, Na, and Mg panel and training the data with classification tree and naïve Bayes, providing values of correct assignment of 68% and 73%, respectively, when comparing AMD and control groups, the percentage of which represents the correctly classified predictions. The obtained area under the curve reached 0.70 to 0.86 when using classification tree and naïve Bayes, respectively.

**Table 3.** Best Classifying Machine-Learning Models Using the Variables MT1A, Na, and Mg, When Comparing AMD Patients and Control Subjects

Method	CA	AUC	Sensitivity	Specificity	Precision
Random forest	0.63	0.73	0.82	0.41	0.61
Naive Bayes	0.73	0.86	0.58	0.90	0.87
k-Nearest Neighbors	0.54	0.59	0.39	0.71	0.60
Classification tree	0.68	0.70	0.69	0.67	0.70
Support vector machine	0.64	0.71	0.77	0.50	0.73

AUC, area under the curve; CA, correct assignment.

## Discussion

This study revealed evidences of a specific altered metal homeostasis in the course of AMD disease, including up-regulation of MT1A and S100A6, and down-regulation of LF, Fe, Mg, and Cu in the tear film of individuals with macular disorder. AMD is characterized by a progressive loss of central vision caused by age-related neurodegenerative changes in the highly specialized region of the central retina responsible for fine and color vision, the macula. During aging, the systemic metabolism of metals, including Fe, Cu, Zn, and Ca, changes, and their homeostatic deregulation may play an important role in the progressive neurodegeneration. Proteins/peptides identified in extracellular deposits characteristic of AMD undergo pathogenic aggregation promoted by metals.^36^^,^^37^ The anomalous protein aggregation observed in AMD may be related to the existence of metal dyshomeostasis, as a cause or consequence.^26^ Hence, the understanding of structural and functional interactions between metals and intracellular and extracellular components becoming pathogens may shed light to the etiology of AMD disorder. Therefore the identification of potential altered levels of metals, metalloids, and proteins interacting with metals in the tear film of AMD patients may contribute to the understanding of the pathophysiological changes observed in this irreversible disorder and, at the same time, propose candidate biomarkers for an early diagnosis or better prognosis of the disease.

We consequently designed a multitargeted analysis based on immunoassays and elemental mass spectrometry for the quantitative determination of LF, S100A6, CFH, CLU, APP, Ca, Mg, P, Na, Zn, Fe and Cu in the tear film of dry AMD patients and control subjects. Tears are produced by the lacrimal apparatus to moisten the ocular surface, protecting the cornea against desiccation, facilitating non-friction-bearing movements of the lids on the globe to improve the refractive effect and preserving against irritation caused by the environment.^38^ The lacrimal glands are classic exocrine acinar glands secreting a dilute aqueous solution containing proteins, small molecular mass components and electrolytes, constituting the tear film. Therefore, the target-biofluid tear film is mainly composed of H_2_O (≈98%), with normal pH = 7.5 and Na, Cl, HCO_3_, K, Mg, Ca, glucose, retinol and urea as major solutes. The most abundant tear proteins include immunoglobulin A, LF, tear-specific prealbumin, G protein, and lysozyme. Also, traces of other proteins, enzymes, and inhibitors from serum origin are present. The normal tear turnover occurs at approximately 16%/min at normal blink rate but is highly dependent on temperature and humidity and can be induced by a variety of mechanical and psychophysical stimuli.^39^^,^^40^ Accordingly, one of the main difficulties of this study deals with the limited sample volume, especially considering the age of the recruited patients. In the case of ELISA assays, tear sample dilution was carried out, permitting the multiparametric quantitation of selected molecules. Also, considering that the multielemental analysis by ICP-MS using conventional nebulization is dramatically limited by high sample volume consumption (up to 200 µL · min^−1^), and even the use of micronebulizers typically permit uptakes of 50 to 100 µL · min^−1^, we decided to optimize and implement the FIA-ICP-MS system, together allowing the quantitation of the 13 selected variables in this biofluid.

Normal composition of human tear has been widely characterized using proteomic,^41^^–^^43^ lipidomic,^44^^–^^46^ metabolomic,^47^ and metallomic^48^ approaches. Proteomic or metabolomic methodologies have been developed for the study of the lacrimal film and the identification of candidate biomarkers in patients diagnosed with glaucoma, dry eye, diabetic retinopathy, keratoconus, or cancer, among others.^19^^,^^25^^,^^49^ However, limited studies addressing the tear film analysis of AMD patients have been published to date. Among them, Yu et al.^50^ observed, using ELISA assays, that the tear (along with serum) of AMD patients (n = 20) had increased IgA titers than control subjects (n = 15), which may imply a role of overactive IgA responses in AMD pathogenesis. Winiarczyk et al.^51^ attempted to define the AMD tear film proteome by MALDI-TOF/TOF using Schirmer strips for sampling in eight patients with wet AMD, six patients with dry AMD, and eight control individuals. From the identification of 342 proteins, shootin-1, histatin-3, fidgetin-like protein 1, SRC kinase signaling inhibitor, Grave's disease carrier protein, actin cytoplasmic 1, prolactin-inducible protein 1 and protein S100-A7A were found up-regulated in the tear film of dry AMD patients, involving specific pathways related to oxidative stress, inflammation, and proteolysis. Recently, the tear film of 15 patients with wet AMD and 15 age-matched healthy controls was comparatively analyzed by two-dimensional gel electrophoresis and MALDI-TOF/TOF, which identified altered proteins belong to pathways involving oxidative stress, protein clearance and chronic inflammation pathways, highlighting annexins A1 and A4 are part of the calcium-dependent phospholipid-binding family that regulate inflammation and autophagy.^52^^,^^53^ However, none of the identified altered proteins in those works coincide with those evaluated in our study showing significant variations.

The significant up-regulation of S100A6 protein observed in the tear film of our AMD group can be related to its role as Ca homeostasis regulator. The S100A6 belongs to the S100 family of Ca^2+^-binding proteins of the EF-hand type (i.e., with conserved calcium-binding motif).^54^ In the eye, S100A6 is expressed in adult corneal endothelial cells but not in the fetal corneal endothelial cells.^55^ The protein levels of S100A6 were previously observed up-regulated in dry eye disease and Meibomian gland dysfunction^32^^,^^56^ and down-regulated in the tear film of patients diagnosed with keratoconus^57^ and were therefore proposed as candidate biomarkers of these ocular surface disorders. Interestingly, S100A6 was found to be up-regulated in experimental corneal neovascularization models^58^ and expressed in pterygium tissue removed from patient,^59^^,^^60^ but no studies related this protein with AMD disease to date.

For the first time, LF down-regulation was observed in AMD, when comparing patients with sex- and age-matched healthy subjects. This glycoprotein is normally presented in most mucosal secretions (i.e. nasal secretion, saliva, etc., in tears, vitreous humor, RPE, and retina).^61^^–^^63^ LF regulates iron homeostasis^64^ and also presents anti-inflammatory, antibacterial, reactive oxygen species modulator, antiviral, and antitumor immunity effects.^65^ This protein has been previously proposed as a candidate biomarker of dry eye disease,^66^ in which oral LF administration preserves lacrimal gland function in aged mice, reducing the oxidative damage and inflammation.^67^ The administration of LF also reduces the choroidal neovascularization in a laser-induced mouse model that mimics the AMD wet form through the inhibition of hypoxia-inducible factors.^68^ Notably, LF concentration in the tear film of control individuals seems to remain invariant through age and independently of sex,^62^^,^^69^ as the current study shows. Moreover, the lower levels of LF observed in the tear film of AMD patients may be related to lower Fe concentration and increased oxidative stress observed during the course of the disease.

The MT1A subisoform belongs to the superfamily of metallothioneins, cysteine rich and metal-binding low molecular mass proteins (<7 kDa), involved in cellular Cu and Zn homeostasis, metal detoxification, defense against oxidative damage through free radical scavenging and neuroprotection.^70^^–^^72^ The MT1A, codified by the *MT1A* gene and expressed in all the human ocular tissues, was observed up-regulated in the tear of AMD patients, highlighting the high variability in this group in contrast to the control subjects (see Fig. 2F). Conversely, previous studies showed that levels of metallothioneins decreased with age in isolated retinal pigmented cells from postmortem donors with signs of AMD, but without determining individual subisoforms.^73^ Remarkably, studies carried out using in vivo and in vitro models of retinal damage showed overexpression of MT1 and MT2 isoforms, suggesting that their up-regulation may be protective against acute retinal insults,^74^^–^^76^ which may explain the higher levels of MT1A observed in the AMD patients of this work.

From the different studies published so far, age-related changes of the tear composition have been observed, including increased expression of proinflammatory cytokines and matrix remodeling factors^77^ and higher plasma-derived albumin levels.^78^ Considering that aging is one of the main risk factors for AMD onset, we additionally studied possible correlation between targeted proteins and age in our cohort, without observing any age-related change in the tear composition, with the exception of APP protein. A negative correlation was obtained for APP, but only in the AMD group (*r* = −0.523, *P* < 0.01), indicating that the levels of this protein decreased with age in AMD patients but not in healthy controls. This observation may be associated with the age-related increase of the activity of APP cleaving enzymes, which contributes to APP degradation and the increased production and accumulation of extracellular deposits,^79^ being markedly declined in the AMD disease group. Consequently, the follow-up of this protein in the tear film of AMD patients during development and advanced stages of the disease warrants additional research.

Current literature does not include any study tackling the multielemental analysis of tear film of AMD patients. We solely identified limited publications attempting to quantify several elements in the tear film by different sampling and analysis approaches. A seminal work quantified the levels of Zn in tears of young healthier volunteers by atomic absorption spectrometry, obtaining a mean value of 1.537 ± 0.146 µg · mL^−1^.^80^ A multielemental analysis of Na, K, Mg, and Ca was carried out in the tear film of 10 healthy humans by ICP-MS or ICP atomic emission spectroscopy, with levels of 104.5 ± 4.27, 18.1 ± 1.04, 0.49 ± 0.02, and 0.33 ± 0.03 mM, respectively.^81^ The multielemental analysis of tears from 60 volunteers from different geographic origins (urban vs rural areas), by ICP-MS, provided averaged concentrations of 18.40 ± 16.53 ng · mL^−1^ for Cu and 29.61 ± 31.46 ng · mL^−1^ for Zn.^48^ Also, the possible relationship between tear Zn levels and helminths parasitic infection was evaluated in 81 individuals using a colorimetric assay, observing higher levels of this element in the infected population (4.78 µg · mL^−1^) when compared to control subjects (2.42 µg · mL^−1^).^82^ Finally, a multielemental analysis of Cu, Zn, Se, Rb, Ba, Pb, Mn, and Co was carried out by ICP-MS in 47 patients diagnosed with type II diabetes and 50 healthy controls, observing significant differences for Zn, Cr, Co, Mn, Ba, and Pb, with averaged concentration of Zn reaching 66.00 ng · mL^−1^ in diabetic patients and 33.25 ng · mL^−1^ in nondiabetic subjects.^83^

The important variability observed for the above quantitative results of trace elements in tears must be stressed, which differs with our results for Cu and Zn (averaged of 0.16 and 0.36 µg · mL^−1^, respectively). Experimental differences may be attributed to the tear sampling procedure or methodological determination. It seems that sampling using Schirmer strips provided lower levels of Zn and Cu,^48^^,^^82^^,^^83^ when compared to the use of glass microcapillary.^80^^,^^81^ Schirmer strips are routinely used in clinic to determine tear production, where the aqueous fraction of the tear travels further along the strips than non-polar proteins, lipids, or metabolites.^42^ This fact, along with the requirement of a quantitative extraction of the analytes from the strips for further analysis, may contribute to the observed differences in sample composition. In fact, the tear composition is affected by the method used for sample collection,^84^ as discussed for proteins,^85^ metabolites,^26^ and lipids.^44^ The use of microcapillary tubes is less invasive, safer, and avoids reflex tearing, leading to the identification of a huge number of extracellular proteins, when compared to the use of Schirmer strips,^25^ but requires trained personal, which could explain the low number of studies using microcapillary sampling. Also, particular care must be taken with all the methodologies to avoid sample contamination during uptake and pretreatment.

The observed lower levels for Fe, Mg and Cu in the AMD cohort of our study may be related to disease onset or progression. The essential element Fe is the most abundant redox-active metal in the human body,^86^ the local accumulation of which has been implicated in the pathogenesis of neurodegenerative diseases, including AMD.^87^ Specifically, higher content of Fe has been detected in the photoreceptors, RPE and its melanosomes, Bruch's membrane, and extracellular deposits of AMD patients,^87^^–^^90^ where its retinal accumulation may contribute to AMD pathogenesis by inducing oxidative stress and inflammation damage. Interestingly, elemental analysis of aqueous humor showed higher levels of Fe in AMD patients when compared to control subjects.^91^ Nonetheless, according to our results, decreased concentration of Fe in the recruited AMD patients seems to be directly related with the observed lower levels of LF, one of the main iron binding proteins in the tear film, which suggest the underlying interplay between both variables.

Likewise, Cu is an essential element for the visual cycle and photoreceptors survival, the deficiency of which has been associated with morphological changes in the retina and optic neuropathy.^92^ The lower Cu concentration determined in the tear film of our recruited patients diagnosed with AMD is in line with the lower levels of Cu observed in the aqueous humor of individuals with nonexudative AMD.^91^ With aging, increased systemic levels of Cu (blood or serum) was observed,^93^ but the local Cu levels in the RPE and choroid were reduced,^94^ specifically in AMD patients,^95^ reflecting lower levels of this element within the affected eye. On the other hand, Mg is the second most abundant intracellular cation involved in maintenance of cell membrane function, synthesis of nucleic acids, and energy metabolism.^96^ Heesterbeek et al.^97^ determined by ICP-MS that Mg levels were higher in the plasma of AMD patients, but without statistical significance. Recently, Chang et al.^98^ reported that multiple dietary nutrients, including Mg, were associated with decreased risk for neovascular AMD. Considering its physiological role in restoring the blood-brain barrier integrity, Mg deficiency has been associated with age-related neurodegenerative diseases, including amyotrophic lateral sclerosis (ALS) and Alzheimer's or Parkinson diseases.^17^^,^^98^ Specifically, Mg levels were lower in the blood of patients with ALS, when compared to control subjects, similarly to the significantly lower concentrations of this element determined in the tear film of AMD patients.

The higher prevalence of dyslipidemia in the AMD group is in agreement with previous studies, showing that pathogenesis may be related to circulating lipids, local lipid transport, or both.^28^^,^^99^ To discard possible confounders of demographic and clinical conditions, including the risk factor dyslipidemia, in the altered levels of target metals and (metallo-)proteins obtained in the tear film of AMD patients, when compared to control subjects, an additional statistical analysis was carried out. Exclusion of the subjects with dyslipidemia from the multivariate analysis provided similar results, as compared to statistical analysis obtained including individuals with that risk factor, with the exception of Mg, probably because of the individual's reduction in the studied cohort, indicating the low influence of this risk factor on the obtained differences. Interestingly, the multivariate logistic regression analysis confirmed that dyslipidemia did not contribute to the observed differences, and therefore the differences observed for MT1A, S100A6, LF, Fe and Cu are as result of AMD disease.

Additionally, the possible correlations between proteins and elements were also investigated, observing a statistically significant positive trend for Mg when comparing with Na, Cu and P (Figs. 4A–C), for both AMD and control subjects. Conversely, positive correlations for Mg and Ca (Fig. 4D), for Ca and Na (Fig. 4E), and for LF and Cu, were exclusively observed for the AMD group and not for the healthy controls. Notably, LF was the only protein showing high positive correlation with Cu (Fig. 4 D), but not for Fe, suggesting a possible interdependence in AMD disease. The highly positive correlation observed for Mg with Na, P and Ca may suggest an agonist behavior of Mg,^100^ and also for Na and P and for LF and Cu. However, the specific positive interaction in the AMD group needs to be elucidated. Interestingly, the quantitative data obtained for the thirteen variables in both AMD and control groups were used to establish a three-protein-metal panel, consisting of MT1A, Na and Mg. This panel was used to generate machine-learning models (see Table 3), obtaining the classification accuracy when comparing AMD patients versus control subjects, which predicts the disease in the 73% of cases. However, the diagnostic power for predicting AMD is quite low using these variables, and it seems that their alterations in tears are not directly associated between themselves.

Even though this is a comprehensive targeted study on trace metals and (metallo-)proteins in patients with dry AMD, this work should be considered as exploratory. This study is limited by the restricted number of samples used for statistical analysis, which would require additional recruited patients to obtain more than 300 samples for each group to reach adequate power of the tests. Hence, the low number of samples used for classifying groups may affect the discriminating power of the studied panel of MT1A, Na and Mg, using the proposed algorithms. Also, evaluation of tear film composition throughout the day and in the long time would be desirable in future studies to determine intra- and/or inter-daily variations. Finally, issues regarding the relevant correlation of the tear film composition with macular lesions are open, considering that although the tear film is not directly connected with the retina, it can be altered by the partial blood-retinal barrier breakdown in the course of AMD.

The multitargeted approach developed for the analysis of limited volume from the tear film permitted us to obtain quantitative information for 13 variables, including LF, S100A6, CFH, CLU, APP, MT1A, Ca, Mg, P, Na, Cu, Fe, and Zn, combining spectrophotometry and mass spectrometry analytical techniques. The specific altered levels of metals and (metallo-)proteins (i.e., MT1A, S100A6, LF, Fe, Mg and Cu) in the tear film of AMD patients suggest compromised metal homeostasis during the progression of this neurodegenerative disease and may contribute to shedding light into the pathophysiology of macular degeneration. The possible relation of observed pathophysiological changes with the anomalous protein aggregation deserves further research.

## Supplementary Material

Supplement 1
