# Supplementary material for: Targeted Analysis of Tears Revealed Specific Altered Metal Homeostasis in Age-Related Macular Degeneration
Source: Invest Ophthalmol Vis Sci. 2022 Apr 15;63(4):10. doi: 10.1167/iovs.63.4.10 (PMC9034717; doi:10.1167/iovs.63.4.10)
Supplement: Supplement 1 [file iovs-63-4-10_s001.pdf]

# *Supplementary Material*

# Targeted analysis of tears revealed specific altered metal homeostasis in age-related macular degeneration

**Eva Valencia[1,2], Montserrat García[2,3], Beatriz Fernández-Vega[2,3], Rosario Pereiro[1,3], Lara Lobo[1]*, Héctor González-Iglesias[2,3,¥*]**

[1]Ophtalmological Research Foundation (University Institute Fernández-Vega, University of Oviedo), 33012 Oviedo, Spain.

[2]Department of Physical and Analytical Chemistry, Faculty of Chemistry, University of Oviedo, Avda. Julián Clavería 8, 33006 Oviedo, Spain.

[3]Ophthalmological Institute Fernández-Vega, Avda. Dres. Fernández-Vega, 34, 33012 Oviedo, Spain.

[¥]Current Address: Instituto de Productos Lácteos de Asturias, Consejo Superior de Investigaciones Científicas (IPLA-CSIC), Villaviciosa, Spain.

*Corresponding authors. Ophthalmological Institute Fernández-Vega, Avda. Dres. Fernández-Vega, 34, 33012, Oviedo, Spain. Telephone: +34985240141. E-mail addresses: lobolara@uniovi.es (L. Lobo), h.gonzalez@fio.as (H. González-Iglesias)

**Keywords: Age-related macular degeneration, tear, targeted analysis, metals, dyshomeostasis.**

**Supplementary Material Description:** The supplementary material contains supplementary data on molecular assays using ELISA kits, the supplementary Table S1 of Analytical parameters of individual ELISA analysis, the Supplementary Table S2 describing common oral treatments in both control subjects and AMD

patients, and the Supplementary Table S3 of Concentrations of proteins and elements determined in the tear film of AMD patients and control subjects without considering patients with dyslipidemia.

**Molecular assays using ELISA kits**

Commercially available sandwich-type ELISA kits from Abcam (UK) were used for the quantification the proteins human lactoferrin (LF, ab200015), human complement factor H (CFH, ab252359), human clusterin (CLU, ab174447) and human amyloid precursor protein (APP, ab216944). Commercial ELISA kits purchased from Abyntek Biopharma (Vizcaya, Spain) and LS BIO (WA, USA) were used for the analysis of human S100 calcium binding protein A6 (S100A6, ABK1-E1028) and human metallothioneins 1A (MT1A, LS-F10296), respectively. Quantitation was performed according to the instructions of the manufacturer, as follows:

Abcam ELISA kit protocol for LF, CFH, CLU and APP. 50 µL of standard or samples were introduced in each well, then antibody cocktail was added. The plate was incubated at room temperature for an hour while was stirred. Afterwards, three washing steps were carried out using 1x PBS Buffer. Finally, a TMB solution was added and after stirring for 10 minutes, the reaction was stopped using a stop solution. Absorbance was measured at 450 nm wavelength.

Abyntek ELISA kit protocol for S100A6. 100 µL of each solution, standard or sample, were added to wells. Then, an incubation step of 1 h at 37oC was carried out. Next, a detection reagent Ab solution was introduced in each well. After 1 h at the same temperature as first incubation step, the plate was washed three times using a 1x PBS Buffer. Then, a second detection reagent Ab solution was added and incubated for 30 min at 37oC. Five washing steps were carried out. A substrate solution was introduced inside all wells and the plate was incubated 10 min at 37oC. Finally, the enzymatic reaction was stopped, and the absorbance was monitored at 450 nm wavelength.

LSBIO ELISA kit protocol for MT1A. 100 µL of each solution, standard or sample, were added to wells. Then, an incubation step of 2 h at 37oC was carried out. Next, a detection reagent Ab solution was introduced

in each well. After 1 h at the same temperature as first incubation step, the plate was washed three time using a 1x PBS Buffer. Then, a second detection reagent Ab solution was added and incubated for 1h at 37 oC. Five washing steps were carried out. A substrate solution was introduced inside all wells and the plate was incubated 10 min at 37oC. Finally, the enzymatic reaction was stopped, and the absorbance was monitored at 450 nm wavelength.

The analytical parameters of each individual ELISA assays are shown in Table S1.

**Table S1.** Analytical parameters of individual ELISA analysis

| ELISA assay | LoD (ng/mL) | LoQ (ng/mL) | Calibration curve | Correlation coefficient | Detection range (pg/mL) |
|---|---|---|---|---|---|
| LF | 0.193 | 0.644 | y = 0.00006x + 0.0667 | 0.998 | 0-10000 |
| CLU | 0.312 | 1.042 | y = 0.00007x + 0.0349 | 0.998 | 0-15000 |
| APP | 0.134 | 0.446 | y = 0.0001x + 0.0538 | 0.998 | 0-6000 |
| CFH | 0.260 | 0.869 | y = 0.00006x + 0.0466 | 0.997 | 0-10000 |
| MT1A | 0.007 | 0.025 | y = 0.0011x + 0.0891 | 0.998 | 0-400 |
| S100A6 | 0.274 | 0.914 | y = 0.0001x + 0.2518 | 0.991 | 0-5000 |

LoD, detection limit; LoQ, quantitation limit

**Study subjects**

**Table S2**. Common oral treatments in both control subjects and AMD patients.

| Oral treatments | Study population (n) | | | |
|---|---|---|---|---|
| | Controls (29) | % controls | AMD (31) | % AMD |
| Lyrica (anticonvulsant) | 2 | 7 | 0 | 0 |

| | | | | |
|---|---|---|---|---|
| Adiro 100 (acetylsalicylic acid) | 3 | 10 | 2 | 6 |
| Enalapril (anti- HTA) | 1 | 3 | 1 | 3 |
| Tramadol (pain) | 1 | 3 | 1 | 3 |
| Sintrom (acenocoumarol) | 2 | 7 | 1 | 3 |
| Omeprazole | 3 | 10 | 3 | 10 |
| Noctamid (lormetazepam) | 2 | 7 | 0 | 0 |
| Orfidal (benzodiazepines) | 2 | 7 | 4 | 13 |
| Hemovsas (pentoxifilina) | 2 | 7 | 0 | 0 |
| Avidart (dutasteride) | 1 | 3 | 1 | 3 |
| Atorvastatin | 1 | 3 | 3 | 10 |
| Tranxilium (dipotassium clorazepate) | 1 | 3 | 1 | 3 |
| Eutirox (levothyroxine) | 1 | 3 | 1 | 3 |
| Diovan (anti-HTA) | 9 | 31 | 11 | 35 |
| Simvastatin | 2 | 7 | 10 | 32 |

**Elemental and molecular quantitation in the tear film of AMD patients and control subjects without dyslipidemia**

**Table S3.** Concentrations of proteins and elements determined in the tear film of AMD patients (n=17) and control subjects (n=27), after filtering dyslipidemic individuals.

Protein Concentrations

| | Control | AMD | Fold-change | p-value |
|---|---|---|---|---|
| Lactoferrin (average ± StD, mg/mL) | 11.96 ± 4.13 | 5.89 ± 3.13 | 0.5 | 0.0005 |
| S100A6 (average ± StD, ng/mL) | 393. 84 ± 153.38 | 541.37 ± 238.47 | 1.4 | 0.04 |
| CFH (average ± StD, ng/mL) | 1632.82 ± 1152.43 | 2047.47 ± 1375.23 | 1.3 | 0.4 |
| Clusterin (average ± StD, mg/mL µg/mL) | 28.67 ± 12.78 | 35.55 ± 15.15 | 1.2 | 0.2 |
| APP (average ± StD, ng/mL) | 174.65 ± 78.86 | 156.03 ± 79.25 | 0.9 | 0.2 |
| MT1A (average ± StD, ng/mL) | 154.31 ± 37.63 | 328.45 ± 258.53 | 2.1 | 0.002 |

| Element Concentrations | | | | |
|---|---|---|---|---|
| Na (average ± StD, µg/mL) | 2141.09 ± 531.66 | 1872.65 ± 864.13 | 0.9 | 0.3 |
| Mg (average ± StD, µg/mL) | 9.748 ± 3.76 | 6.81 ± 4.16 | 0.7 | 0.07 |
| P (average ± StD, µg/mL) | 3.96 ± 2.31 | 3.36 ± 2.80 | 0.8 | 0.6 |
| Ca (average ± StD, µg/mL) | 22.26 ± 7.38 | 22.67 ± 16.66 | 1.0 | 0.8 |
| Fe (average ± StD, µg/mL) | 3.22 ± 1.88 | 1.75 ± 1.00 | 0.5 | 0.02 |
| Cu (average ± StD, µg/mL) | 0.16 ± 0.06 | 0.10 ± 0.04 | 0.6 | 0.01 |
| Zn (average ± StD, µg/mL) | 0.36 ± 0.27 | 0.32 ± 0.22 | 0.9 | 0.7 |
